# Growth promotion and protection from drought in *Eucalyptus grandis* seedlings inoculated with beneficial bacteria embedded in a superabsorbent polymer

**DOI:** 10.1038/s41598-020-75212-4

**Published:** 2020-10-26

**Authors:** José María Chaín, Esteban Tubert, Corina Graciano, Luis Nazareno Castagno, Marina Recchi, Fernando Luis Pieckenstain, María Julia Estrella, Gustavo Gudesblat, Gabriela Amodeo, Irene Baroli

**Affiliations:** 1 Departamento de Biodiversidad y Biología Experimental, Facultad de Ciencias Exactas y Naturales, Universidad de Buenos Aires, Buenos Aires, Argentina; 2 Instituto de Biodiversidad y Biología Experimental y Aplicada (IBBEA), Consejo Nacional de Investigaciones Científicas y Técnicas (CONICET) - Universidad de Buenos Aires, Buenos Aires, Argentina; 3 Instituto de Fisiología Vegetal (INFIVE), Consejo Nacional de Investigaciones Científicas y Técnicas (CONICET) - Universidad Nacional de La Plata, La Plata, Argentina; 4 Instituto Tecnológico de Chascomús (INTECH), Consejo Nacional de Investigaciones Científicas y Técnicas (CONICET) - Universidad Nacional de San Martín (UNSAM), Chascomús, Argentina; 5 Present Address: Departamento de Fisiología, Biología Molecular y Celular “Profesor Héctor Maldonado”, Instituto de Biociencias, Biotecnología y Biología Translacional (IB3), Facultad de Ciencias Exactas y Naturales, Universidad de Buenos Aires, Buenos Aires, Argentina; 6 Present Address: Consejo Nacional de Investigaciones Científicas y Técnicas (CONICET), Godoy Cruz 2290, C1425FQB Buenos Aires, Argentina

**Keywords:** Microbiology, Plant sciences, Materials science

## Abstract

*Eucalyptus grandis* is a globally important tree crop. Greenhouse-grown tree seedlings often face water deficit after outplanting to the field, which can affect their survival and establishment severely. This can be alleviated by the application of superabsorbent hydrophilic polymers (SAPs). Growth promoting bacteria can also improve crop abiotic stress tolerance; however, their use in trees is limited, partly due to difficulties in the application and viability loss. In this work, we evaluated the improvement of drought tolerance of *E. grandis* seedlings by inoculating with two *Pseudomonas* strains (named M25 and N33), carried by an acrylic-hydrocellulosic SAP. We observed significant bacterial survival in the seedling rhizosphere 50 days after inoculation. Under gradual water deficit conditions, we observed a considerable increase in the water content and wall elasticity of M25-inoculated plants and a trend towards growth promotion with both bacteria. Under rapid water deficit conditions, which caused partial defoliation, both strains significantly enhanced the formation of new leaves, while inoculation with M25 reduced the transpiration rate. Co-inoculation with M25 and N33 substantially increased growth and photosynthetic capacity. We conclude that the selected bacteria can benefit *E. grandis* early growth and can be easily inoculated at transplant by using an acrylic-hydrocellulosic SAP.

## Introduction

Planted forests, which have increased in area an average of 4.4 million ha per year in the period 1990–2015^1^, are a major source of timber and raw materials for paper and biofuel production. Their existence helps to reduce harvesting from natural forests globally, besides providing a considerable CO_2_ sink. In subtropical and tropical regions, the highly productive *Eucalyptus grandis* is the most widely planted fast-growing species^2^. Because water availability is the most important environmental factor limiting plant growth and development, the productivity of forest ecosystems is severely constrained by water deficit^3^. Thus, because the eucalypt growing regions are expected to undergo severe drought in future climate scenarios^4^, it is important to develop ways to maximize water use efficiency and drought tolerance in this species, either through management or by breeding new, more efficient germplasm.

In planted forests, the seedling establishment stage is particularly sensitive to water deficit, due to the high evaporative demand in cleared areas with high solar radiation, complicated by the fact that seedlings rely exclusively on precipitations and on the water retention properties of the upper layer of the soil. Moreover, low soil water potentials have a negative impact on root elongation, which makes drought an important impediment for seedling survival immediately after plantation. Thus, rapid root growth is essential for implantation success, to ensure access to water and nutrients, consequently allowing for vigorous shoot development and successful competition with other plants for light^5^. To maintain proper water content in the seedling rhizosphere during establishment, producers usually add water-saving hydrogels (superabsorbent polymers or SAPs) at the time of planting^6^. These polymers are highly hydrophilic matrixes that can incorporate up to 600% of their weight of water^7^. Different SAP properties, such as viscosity, water retention, toughness, and degradability can be achieved by combining monomers and by altering the crosslinking status^8^. Highly crosslinked acrylic-based SAPs have been shown to be very effective at reducing post-planting water deficit stress at an affordable cost and simple field application methodologies have been developed^9–11^. In particular, an acrylic-cellulosic superabsorbent composite (SAPH) has been employed in *E. grandis* seedlings in greenhouse trials, as well as in the field, and shown to be effective not only for water retention but also for nutrient delivery^12^.

The susceptibility of a plant to drought and the deployment of tolerance strategies depend on the plant species, growth stage and the severity of the stress. In general drought tolerance is achieved by minimizing water loss and/or maximizing water uptake^13^. In the short term, plants respond to water deficit by decreasing transpiration through control of stomatal conductance, which can provoke carbon limitation for photosynthesis, decreasing net CO_2_ assimilation^14–17^. Under severe or prolonged drought, shedding of leaves also leads to a reduced transpiration rate at the whole plant level. These strategies are common in many *Eucalyptus* species^15,18–20^ but have the disadvantage of causing slow growth and a substantial reduction in productivity. Another strategy observed in eucalypt seedlings under moderate drought stress is an increase in biomass allocation to the roots to maximize water uptake^14,18^. Elastic and osmotic adjustments also enable plants to sustain cell expansion and maintain leaf water content under drought. Thus, photosynthesis and growth are higher in plants that can make osmotic or elastic adjustment in response to drought. At the cellular level eucalypts, and in particular *E. grandis*, maintain cell turgor under water deficit by the active accumulation of organic solutes, such as proline, and by adjusting the elasticity of cell walls^15,17,19,21,22^.

Plants can host communities of microorganisms, especially bacteria, inside and on the surface of roots and aerial organs. Some of these bacteria can be beneficial for the plant and thus are known as plant growth-promoting bacteria (PGPB;^23^). PGPB promote plant growth in a direct manner through different activities and mechanisms, such as phytohormone production, nitrogen fixation, phosphate solubilization and iron chelation by siderophore production, or indirectly through activities such as suppression of pathogen growth or induction of pathogen resistance^24–26^. Besides growth promotion under optimal conditions, there is an ever-growing number of reports showing that inoculation with PGPB can improve tolerance to abiotic stresses (e.g., drought, heat, and salinity) in crops^23,27–29^. Under water deficit, PGPB have been shown to elicit an induced systemic tolerance response involving hormone regulation. This may entail the release of indole acetic acids that change root architecture or of abscisic acid, which alleviates abiotic stress by provoking early stomatal closure or by modulation of ethylene signaling, leading to delayed tissue senescence. Inoculation with PGPB increases the relative water content of leaves in many plant species and affects other physiological parameters such as chlorophyll content, nutrient uptake, photosynthetic rate and water use efficiency^30,31^. The positive effect of PGPB sometimes may be evident only during drought or after drought recovery, and not under well-watered conditions^32,33^. On the other hand, strains of PGPB that confer drought tolerance may also benefit plant growth under other abiotic stresses^34^. Moreover, PGPB usually do not show host specificity and can colonize and exert their beneficial effects in multiple plant species^31^. A synergistic beneficial effect is frequently observed when bacterial strains are inoculated together in a consortium, independently of the number of strains that compose the consortium^31,35^. Thus, the use of PGPB is rapidly gaining importance as a natural and cost-effective alternative to increase crop yield and manage plant stress in the quest to develop a climate-change resilient agriculture, minimizing the use of synthetic fertilizers and agrochemicals.

Several genera of proteobacteria present in the soil microbiota have been described in relation to their plant growth-promoting ability^36^. *Pseudomonas* is a genus of Gram-negative, non-spore-forming bacteria commonly found in bulk and rhizosphere soil of different types. *Pseudomonas* are quite versatile in their ability to use various substrates as nutrients and can survive under soil conditions that would be stressful for other bacteria^37^. Different strains of *Pseudomonas* have been widely reported as PGPB with a further positive effect on plant drought tolerance^33^. Despite the abundance of scientific reports in which promising PGPB were identified and characterized in the laboratory, many of them have shown limited success in the field^38^. In fact, bacterial populations decline rapidly when they are inoculated directly into the soil without a proper carrier, probably because they must compete with a well-adapted native microbiota and withstand predation. Thus, it is convenient to formulate bacterial inoculants with solid supports that provide physical protection and a suitable microenvironment for bacteria to grow. High quality carriers must sustain consistent viability of the inoculum during formulation, transport, storage and the implantation process. On the other hand, the combination of carrier and selected strains should minimize cost and contamination risks, while being easy to apply and scale-up^39^. Several types of carriers are utilized commercially in the preparation of inoculants. Inert materials, biofilms and liquid formulations are used to coat seeds before sowing^40^; however, these strategies are unavailable to the forest industry, where the starting point of the plantation is a seedling with a developing root system. In commercial plantations, especially of eucalypts, applying a cushion of SAPs into the planting hole to condition the soil and retain water is becoming routine during seedling implantation^6^, so SAPs could be an effective carrier for PGPB. But there are only a few reports of SAPs applied in conjunction with PGPB in the literature, and none of them relates to tree seedlings. For example, it has been shown that there was no negative interaction of SAP and a *Micrococcaceae* strain on the growth of *Arundo donax* under alternating wet and dry conditions^41^. A similar effect was reported on maize inoculated with a combination of *Azospirillum lipoferum* and *Pseudomonas putida* and sown on soil conditioned with SAP, both under a normal watering regime and when subjected to drought^42^.

In this work, we tested, as a PGPB carrier, a crosslinked acrylic-cellulosic superabsorbent composite (SAPH) which we have previously synthesized and shown to be effective for water retention and protection from drought stress and as a matrix for delivery of mineral nutrients when raising *E. grandis* seedlings, both in greenhouse trials and in the field^12^. We selected two bacterial strains with eucalypt growth-promoting characteristics and combined them with gelified SAPH in potted plant experiments in which we tested the performance of the gel-bacteria combination on the plant´s tolerance to two different intensities of drought stress.

## Results

### Selection of bacterial strains

**Figure 1:**
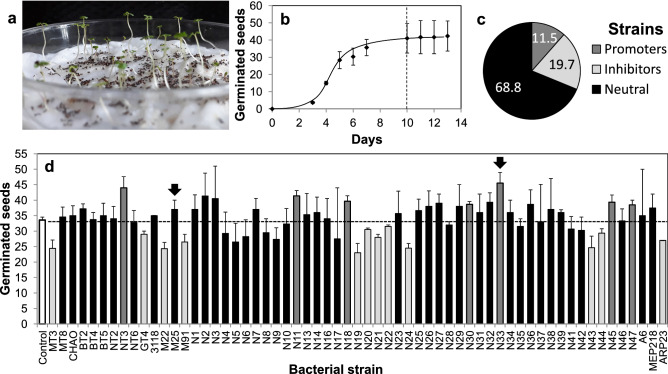
Selection of bacterial strains for their effect on germination. In all cases, we counted the number of germinated *E. grandis* seeds from 50 mg of commercial seed mixture. (**a**) Germination plate ten days after imbibition. (**b**) Germination rate of uninoculated *E. grandis* seeds. The dotted line indicates the tenth day, when the maximum germination is reached. Data shown are the means, n = 3, error bars represent SEM. (**c**) Proportion of tested bacterial strains resulting in promotion, inhibition or no effect on *E. grandis* seed germination. (**d**) Number of emerged seedlings on the tenth day after inoculating seeds with axenic suspensions of different bacterial strains (1 × 10^8^ CFU ml^−1^) or with distilled water (control, white bar). Data shown are the means, the dotted horizontal line indicates the mean of the control treatment. n = 3, error bars represent SEM. Light gray, dark gray and black bars show bacterial strains with negative, positive or no effect on germination, respectively (CI = 95%). Strains M25 and N33 (indicated with arrows) were selected for further studies.

A screening scheme to select PGPB could be applied at different stages of a crop’s life cycle. However, identifying PGPB in tree crops is challenging because of their long life-cycle compared to annual crops. A practical approach is to screen beneficial bacteria for their effect on germination and initial seedling growth^23^, and this is feasible in eucalypts because germination and seedling emergence can be readily triggered by simple imbibition. Thus, to identify strains able to promote growth in *E. grandis* we screened a collection of soil and phyllospheric bacteria associated to different crops^43–47^ for their ability to enhance seed germination rates. Under control conditions (no bacteria added) the number of emerged seedlings reached a plateau (33–40 seedlings from 50 mg of seed mixture) on the tenth day after the start of seed imbibition with distilled water (Fig. 1a,b). We used this parameter to discard strains that reduced the germination frequency. We found that the majority (68.8%) of the strains tested had no significant effect on germination frequency, whereas 19.7% had a significant inhibitory effect and 11.5% enhanced it (confidence interval = 95%; Fig. 1c,d). Strain N33 was selected for its capacity to stimulate seed germination (Fig. 1d). Our analysis of the 16S rRNA gene sequence demonstrated that this strain shares a 99% identity with type strains of the genus *Pseudomonas*. With some of the non-inhibitory strains, we performed nutrition and water management assays. Strain M25, previously identified as a member of the *Pseudomonas* genus by the same methodology^43^ was found to enhance the efficiency of water use in seedlings while N33 showed a growth promotion effect (data not shown). Therefore, these two strains were selected for further studies.

### Survival of bacterial strains in a superabsorbent polymer

**Figure 2:**
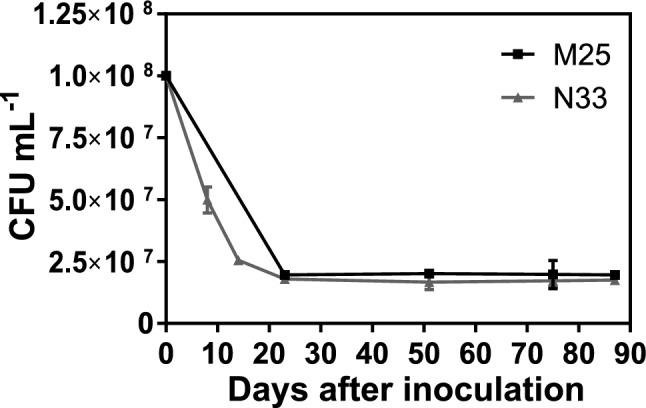
Survival of strains N33 and M25 embedded into a superabsorbent hydrophilic polymer (SAPH). Number of colony-forming units (CFU) recovered per ml of the SAPH-bacteria mixture as a function of time after inoculation. Data shown are the means calculated from duplicated plates of each sample. Error bars indicate the range. If not visible, error bars are smaller than the symbols.

To assess the feasibility of using an acrylic-cellulosic superabsorbent polymer (SAPH^12^) as a vehicle for the application of the selected strains, we tested the survival of bacterial cells when they were embedded in hydrated SAPH. We used cell suspensions of each strain containing 1 × 10^8^ colony forming units (CFUs) per ml in distilled water, while control SAPH was hydrated with distilled water only. Both M25 and N33 showed a gradual one-log decline in the number of viable cells within the SAPH, and a final stabilization approximately 20 days after inoculation when the cells reached a concentration of 1 × 10^7^ CFU ml^−1^ (Fig. 2). Twelve weeks after inoculation we were still able to recover 1 × 10^7^ CFU ml^−1^ of both strains, a titer only one order of magnitude lower than the original inoculum. We thus concluded that the acrylic-cellulosic SAPH is an appropriate carrier for the application of bacterial cells in the root proximity of *E. grandis* seedlings.

### Effect of bacterial inoculation on tolerance to drought in E. grandis seedlings

We generated two different degrees of water deficit stress by manipulating seedling growth conditions and then analyzed the protection conferred by the bacteria. In parallel, we evaluated the promotion of seedling growth under normal watering conditions.

### Effect of individual bacterial inoculation on tolerance to gradual drought in *E. grandis* seedlings

**Figure 3:**
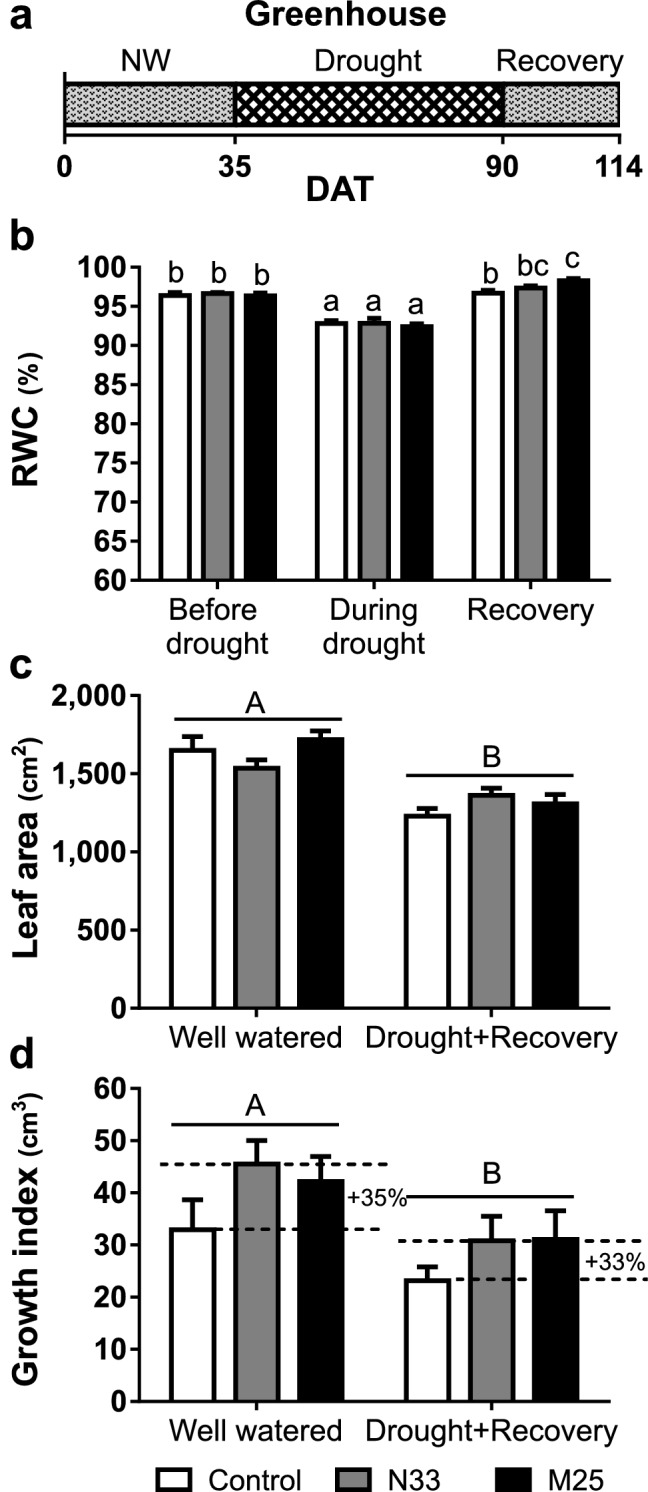
Physiological evaluation of *E. grandis* seedlings subjected to gradual drought in a greenhouse. (**a**) Watering regime for the water deficit experiment. During normal watering (NW) and recovery, the plants were watered to field capacity; during drought, the watering was suspended for 55 days (DAT, days after transplantation). (**b**) Relative water content (RWC) of individual leaves before and during the drought stress, and 24 days after starting the recovery period (35, 90, and 114 DAT, respectively). (**c** and** d**) Growth parameters, computed upon harvesting the plants at the end of the experiment, for well-watered plants and plants subjected to drought and recovery. (**c**) Total leaf area. (**d**) Growth index calculated by subtracting the initial volume index (the product of stem height, *h* and the square of the collar diameter, *d*^2^) from the final volume index for each plant. Dotted lines indicate the differences in mean growth index increment between inoculated plants and the non-inoculated controls for the same water condition. Data shown are the means, n = 12, error bars indicate SEM. Different letters indicate significant differences in a two-way ANOVA (*P*-value < 0.05). Uppercase letters indicate the irrigation factor (well watered, drought or recovery). Lowercase letters indicate the interaction between inoculation (non-inoculated, N33 or M25) and irrigation factors. The inoculation factor was not significant in all cases.

To provide a gradual water deficit stress we grew the *E. grandis* seedlings in large pots (10 l) in a greenhouse, between February and June 2018, in the city of La Plata (− 34.912375, − 57.930637; Province of Buenos Aires, Argentina). Plants were transplanted into pots, and the bacteria added in the planting hole, previously mixed with hydrated SAPH (hydrated SAPH without bacteria was added to control plants). Plants were watered to field capacity every two days for 35 days, at which time watering was stopped in one-half of the pots (“drought” in Fig. 3a), whereas the other half continued to be watered normally (“well-watered” in Fig. 3). Water loss was computed regularly by weighing the pots. Normal watering was resumed after 55 days (when water loss had stabilized at a minimal value) and was maintained for a further 24-day period. Relative water content (RWC) of plants not exposed to water deficit stress was 96%, irrespective of the presence of bacteria in the SAPH matrix (Fig. 3b). Fifty-five days after watering was stopped, all plants showed a ~ 4% decrease in their leaf RWC (*P*-value < 0.05, Fig. 3b). After drought stress and the subsequent 24-day recovery period, seedlings inoculated with M25 had a 2% higher RWC than control ones. The total leaf area and growth index reflected the negative effect of the drought, regardless of the inoculum applied (Fig. 3c,d). We observed that the drought treatment stopped the formation of new leaves, caused slight defoliation and slowed down stem growth (Supplementary Fig. S1). Under the two contrasting watering regimes both bacterial strains brought about a strong positive trend to higher (33–35%) growth indexes, but the differences were not statistically significant (*P*-value > 0.05; Fig. 3d).

**Figure 4:**
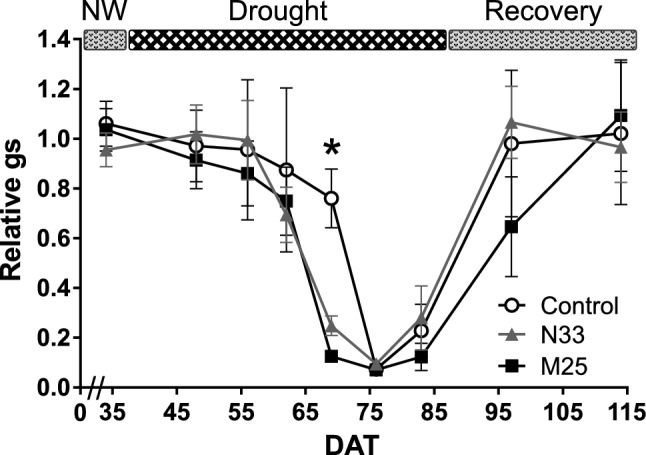
Stomatal conductance (g_s_) of *E. grandis* seedlings subjected to gradual drought stress in a greenhouse. Measurements were performed using a leaf porometer. The irrigation scheme is shown on top of the graph (NW, normal watering). The x-axis represents days after transplantation (DAT), while y-axis shows the average values of g_s_ for stressed and recovered plants, relative to the respective well-watered control. Error bars indicate propagated SEM. Asterisk indicates significant differences between control and inoculated plants in a one-way ANOVA (*P*-value < 0.05).

The time response of stomata to water deficit showed a biphasic behavior (Fig. 4). In non-inoculated plants, stomatal conductance decreased gradually for approximately 30 days since the suspension of irrigation (between 35 and 70 DAT), reflecting the progressive nature of the drought stress imposed. After that, drought stress caused a pronounced fall in stomatal conductance (g_s_) from 80 to 10%, relative to the well-watered controls. The inoculated plants showed an earlier stomatal response against water deficit and reached their lowest g_s_ values 7 days before the control plants. When the watering was resumed, non-inoculated and N33 plants showed a faster recovery of g_s_ values than M25 inoculated plants. Together with the RWC data, these results suggest a mechanism through which the M25 inoculated plants conserve water, reaching higher values of relative water content (RWC) than non-inoculated plants. This is also consistent with the intermediate RWC response of N33 plants shown in Fig. 3.

Additionally, hydraulic and water management parameters were evaluated by fitting pressure–volume curves from a branch of every plant. The apoplastic RWC of branches was higher (32 to 60%) in plants inoculated with M25, independently of the watering regime. Plants inoculated with N33 displayed an intermediate response between control plants and those inoculated with M25 (Table 1). Similar results were observed when the elasticity coefficient (E_max_) was evaluated (Table 1). There were no changes in osmotic potential neither at water saturation nor at turgor loss point (Ψo saturation and Ψo TLP, respectively), independently of the watering regime or inoculation factor. These results suggest that inoculated plants can modify their water management strategy by altering the properties of the cell wall.

**Table 1:** Shoot hydraulic measurements of *E. grandis* seedlings grown in a greenhouse.

Treatment		Ψo saturation (MPa)		Ψo TLP (MPa)		Apoplastic RWC		E_max_ (MPa)	
Control	Well watered	− 1.32 ± 0.10	NS	− 1.49 ± 0.08	NS	0.40 ± 0.08	A	8.40 ± 1.13	A
	Drought + Recovery	− 1.39 ± 0.04		− 1.53 ± 0.05		0.29 ± 0.04		9.96 ± 0.51	
N33	Well watered	− 1.14 ± 0.08		− 1.33 ± 0.06		0.52 ± 0.09	AB	6.78 ± 1.20	AB
	Drought + Recovery	− 1.29 ± 0.11		− 1.47 ± 0.09		0.45 ± 0.09		7.77 ± 1.30	
M25	Well watered	− 1.15 ± 0.09		− 1.35 ± 0.08		0.53 ± 0.08	**B**	6.56 ± 1.09	**B**
	Drought + Recovery	− 1.13 ± 0.05		− 1.38 ± 0.03		0.61 ± 0.07		5.42 ± 0.99	

Plants were cultivated under two water conditions: “Well watered” as a regular watering or “Drought + Recovery”, consisting of watering for 35 days, then suspending the irrigation for a period of 55 days, followed by 24 days with watering at field capacity. Ψo saturation = osmotic potential at inner water saturation; Ψo TLP = osmotic potential at the turgor loss point; apoplastic RWC = relative water content in the apoplast; E_max_ = coefficient of maximum elasticity. Measurements were made in a branch excised from each of 6 plants per treatment. Results are expressed as means ± SEM. Different letters indicate significant differences in a two-way ANOVA (*P*-value < 0.05). NS = non-significant.

### Effect of individual bacterial inoculation on tolerance to rapidly imposed drought in *E. grandis* seedlings

**Figure 5:**
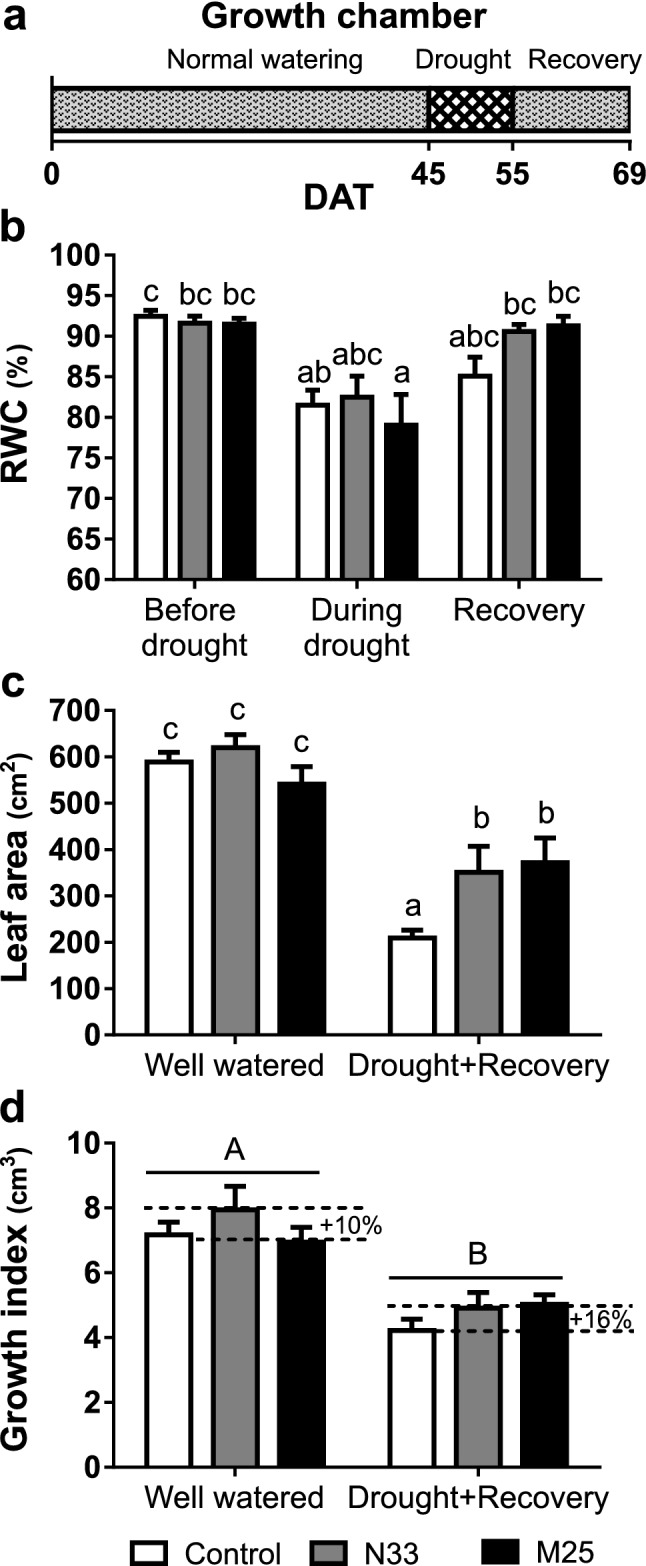
Physiological evaluation of *E. grandis* seedlings subjected to a rapid drought in a growth chamber. (**a**) Watering regime for the water deficit experiment. During normal watering and recovery plants were watered to field capacity; during drought, the watering was suspended for 10 days (DAT, days after transplantation). (**b**) Relative water content (RWC) of individual leaves before and during the drought stress, and 14 days into the recovery period (DAT 45, 55, and 69, respectively). (**c** and** d**) Growth parameters, computed upon harvesting the plants at the end of the experiment, for well-watered plants and plants subjected to drought and recovery. (**c**) Total leaf area. (**d**) Growth index calculated by subtracting the initial volume index (the product of stem height, *h* and the square of the collar diameter, *d*^2^) from the final volume index for each plant. Dotted lines indicate differences in mean growth index increment between inoculated plants and the non-inoculated controls for the same water condition. Data shown are the means, n = 8, error bars indicate SEM. Different letters indicate statistically significant differences in a two-way ANOVA (*P*-value < 0.05). Uppercase letters indicate the irrigation factor (well watered or drought + recovery). Lowercase letters indicate the interaction between inoculation (non-inoculated, N33 or M25) and irrigation factors. The inoculation factor was not significant in all cases.

To assess the protection conferred by the bacterial strains to plants subjected to a more rapid and pronounced water deficit stress than in the greenhouse, we analyzed *E. grandis* seedlings (median height = 14 ± 3 cm) grown in small pots (0.5 l) in a controlled environment chamber to produce faster soil dehydration. Water stress was imposed on half the plants 45 days after transfer to the pots. The substrate reached its minimum soil water content within 10 days after the withdrawal of watering (data not shown). This drought regime (Fig. 5a) led to a more marked drop in growth parameters than the gradual stress imposed in the greenhouse experiment, causing severe defoliation and a considerable delay in growth, both in control and inoculated plants (compare Figs. 3 and 5). The foliar RWC was 92.5% in all well-watered plants regardless of the presence of inoculated bacteria (Fig. 5b). Ten days after watering was stopped, the leaf RWC had fallen significantly (12–15%; *P*-value < 0.05) in control and M25-inoculated plants. In these two groups, RWC recovered upon rewatering, but only attaining 85% in the uninoculated plants, nearly 7% below the RWC before withdrawing watering, whereas plants treated with strain M25 fully recovered their initial RWC (Fig. 5b). In contrast, plants inoculated with N33 showed no significant response of RWC to the water treatment at any of the three assayed stages, although there was a trend to a lower RWC upon drought. In general, these results mirrored those obtained from the greenhouse experiment.

Under normal watering conditions, none of the plants presented significant differences in the final leaf area. After recovering from drought stress, the plants inoculated with either M25 or N33 showed a significantly higher final leaf area than control plants (*P*-value < 0.05; Fig. 5c). Supplementary Fig. S2 shows changes in total leaf area, estimated periodically from light interception measurements. The drought treatment stopped the formation of new leaves and caused severe defoliation in the three groups of plants. However, inoculated plants presented a higher capacity for maintaining their foliage during drought stress and were able to produce new leaves more rapidly when normal watering was resumed.

In well-watered conditions, the growth index of seedlings inoculated with N33 showed a statistically non-significant but positive trend towards higher values relative to non-inoculated plants (approximately 10%; Fig. 5d). Plants subjected to drought showed a statistically significant drop in growth index independently of inoculation, and treatment with both strains caused a positive trend in growth, resulting in a 16% higher growth index (Fig. 5d).

For the same growth period, we observed a higher growth index in the greenhouse assay than in the chamber assay (Supplementary Fig. S3), which can be attributable to differences in the span of the experiments and growth conditions. Eucalypts in the greenhouse had a more favorable light intensity and 20-fold larger pots than those grown in the chamber. The rapidly imposed drought had a more marked effect on root and leaf final biomass than the gradual drought. In both experiments, the effect of drought was significant (*P*-value < 0.05) on stem final biomass, but no differences were observed with bacterial inoculation. The root-to-shoot biomass ratio did not substantially change in response to water treatment or bacterial presence (Supplementary Fig. S1).

**Figure 6:**
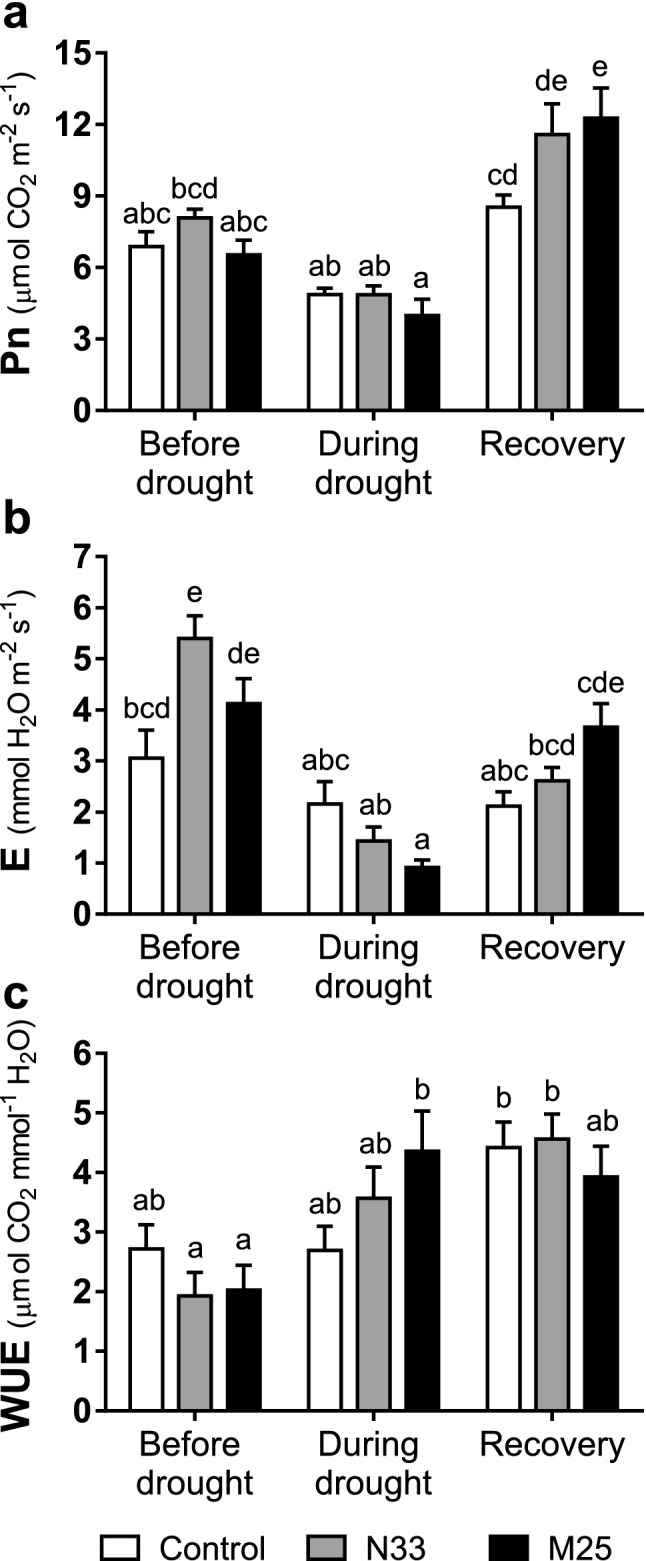
Evaluation of gas exchange parameters from *E. grandis* seedlings subjected to rapid drought stress. Measurements were performed with an infrared gas analyzer at three different times: 45 DAT (before imposing the drought stress), 52 DAT (after 7 days without watering) and 69 DAT (recovery period, 14 days after the resumption of normal watering). The intrinsic water use efficiency (WUE) was calculated by dividing net photosynthesis (P_n_) and transpiration rate (E). Data shown are the means, n = 8, error bars indicate SEM. Lowercase letters indicate the interaction between inoculation (non-inoculated, N33 or M25) and irrigation (before drought, during drought or recovery) factors. Different letters indicate statistically significant differences in a two-way ANOVA (*P*-value < 0.05).

All plants in the growth chamber were evaluated in their net photosynthetic (P_n_) and transpiration (E) capacity with an infrared gas analyzer after leaf acclimation under saturating light. Measurements were performed at 45, 52 and 69 DAT on the youngest mature leaf from the main stem in each plant. Figure 6a shows the stimulation of P_n_ under normal watering caused by inoculation with N33 but not with M25. In all plants, the 7-day withdrawal of watering led to a 30–40% drop in P_n_, independent of preceding inoculation. Net photosynthesis recovered to values above the initial ones in all plants 14 days after resuming watering. Compared to the non-inoculated controls, inoculated plants showed higher P_n_ after recovery, those inoculated with M25 showed a significant P_n_ increase of as much as 40% (*P*-value < 0.05). The increase in P_n_ observed in well-watered plants inoculated with N33 was accompanied by an increase in E (Fig. 6b), as a consequence, their intrinsic water use efficiency (WUE = P_n_ E^−1^) was not significantly different from that of non-inoculated plants (Fig. 6c). After seven days without watering control plants still maintained a relatively high E, whereas plants inoculated with either strain decreased it markedly, consistently with the higher reduction in g_s_ in inoculated plants than in control plants observed under drought in the greenhouse experiment (Fig. 4). This decrease was more pronounced in M25-inoculated plants, which showed the highest intrinsic WUE during the drought of all the groups tested. In all plants, the highest P_n_ values attained upon recovery were not associated with proportional increases in E, and as a result, the plants showed a higher intrinsic WUE than at the onset of drought, without statistical differences between inoculated and non-inoculated plants.

### Effect of bacterial dual inoculation on tolerance to rapidly imposed drought in *E. grandis* seedlings

**Figure 7:**
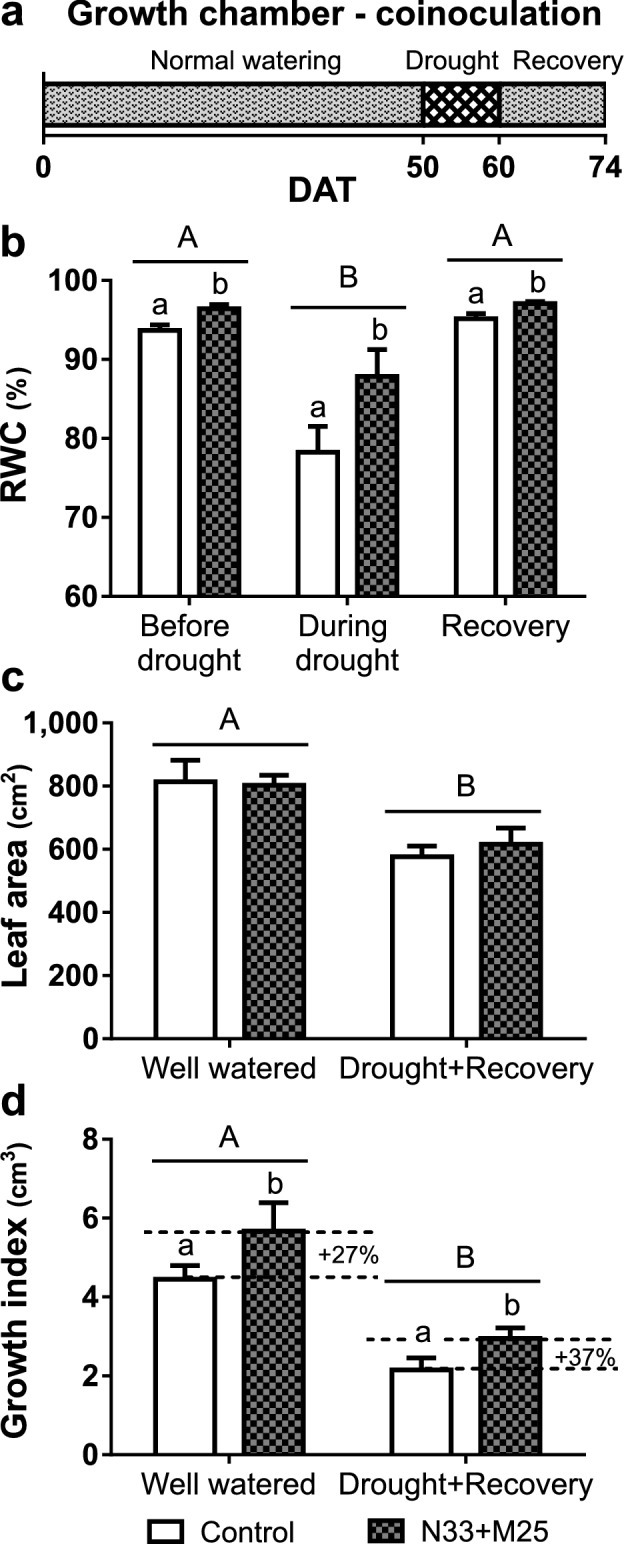
Physiological evaluation of *E. grandis* seedlings co-inoculated with N33 and M25 and subjected to a rapid drought in a growth chamber. (**a**) Watering regime for the water deficit experiment. During normal watering and recovery, the plants were watered to field capacity; during drought, the watering was suspended for 10 days (DAT, days after transplantation). (**b**) Relative water content (RWC) of individual leaves before and during the drought stress, and 14 days into the recovery period (DAT 50, 60, and 74, respectively). (**c** and **d**) Growth parameters, measured upon harvesting the plants at the end of the experiment, for well-watered plants and plants subjected to drought and recovery. (**c**) Total leaf area. (**d**) Growth index calculated by subtracting the initial volume index (the product of stem height, *h* and the square of the collar diameter, *d*^*2*^) from the final volume index for each plant. Dotted lines indicate differences in mean growth index increment between inoculated plants and the non-inoculated controls for the same water condition. Data shown are the means, n = 10, error bars indicate SEM. Different letters indicate significant differences in a two-way ANOVA (*P*-value < 0.05). Uppercase letters indicate the irrigation factor (well watered, drought or recovery), while lowercase letters indicate the inoculation factor (non-inoculated or N33 + M25). The interaction between irrigation and inoculation factors was no significant in all cases.

To assess the possibility of interactions between the two bacterial strains in the protection against water deficit stress, we performed a variation of our previous growth-chamber rapid drought assay. Eucalypt seedlings were inoculated at transplantation time with equal CFU numbers of N33 and M25, reaching the same total cell concentration as used in the single-inoculum experiments. Water stress was imposed on half the plants 50 days after transfer into the pots when plants had reached the same size as in the previous growth-chamber assay. The substrate reached its minimum soil water content within 10 days after the withdrawal of watering (data not shown). A 14-day recovery period under normal watering followed the drought stress, completing a total experiment duration of 74 days (Fig. 7a). In this experiment, the foliar RWC was 3% higher (*P*-value < 0.05) in co-inoculated seedlings than in non-inoculated ones, even before imposing the drought stress. Ten days after watering was stopped, the leaf RWC fell substantially but to a lesser extent in co-inoculated (12%) than in control plants (22%). RWC recovered upon re-watering, reaching similar values to those detected before drought stress, and maintaining the differences between non-inoculated and co-inoculated plants (Fig. 7b). The drought treatment halted the formation of new leaves and caused severe defoliation, but there were no differences between non-inoculated and co-inoculated plants (Fig. 7c). The co-inoculation with N33 and M25 considerably promoted growth, irrespective of the irrigation regime. Compared with non-inoculated plants, the co-inoculated seedlings showed 27 and 37% higher growth index under well-watered conditions and after recovery from drought stress, respectively (Fig. 7d).

**Figure 8:**
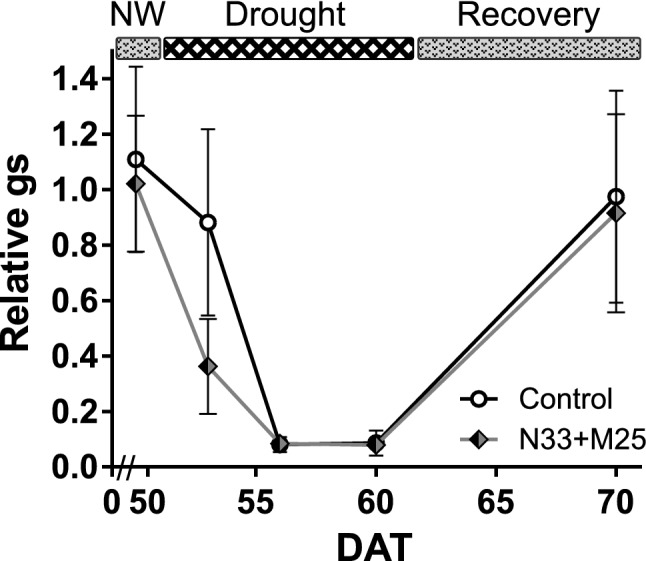
Stomatal conductance (g_s_) of *E. grandis* seedlings grown in a growth chamber and co-inoculated with both bacterial strains. The measures were taken with a leaf porometer. The graph shows the time between the last measurement before imposing the drought stress and the end of the assay. The irrigation scheme is shown on top of the graph (NW, normal watering). Days after transplant (DAT) are shown on the x-axis while y-axis shows the average values of g_s_ for stressed and recovered plants, relative to their well-watered control. n = 8, error bars represent propagated SEM. If not visible, error bars are smaller than the symbols.

As in the previous experiments, we monitored water management by measuring the stomatal conductance of inoculated and control plants before, during and after recovering from drought. The stomatal conductance of droughted plants rapidly decreased from 100 to 10%, relative to the well-watered controls. This occurred in less than ten days after the suspension of irrigation (between 50 and 60 DAT), reflecting the rapid nature of the drought stress imposed. However, and similar to previous experiments, the drop in g_s_ of co-inoculated plants presented a steeper initial slope than in control plants. When watering was resumed, all plants recovered g_s_ values equivalent to those shown before the onset of drought (Fig. 8).

**Figure 9:**
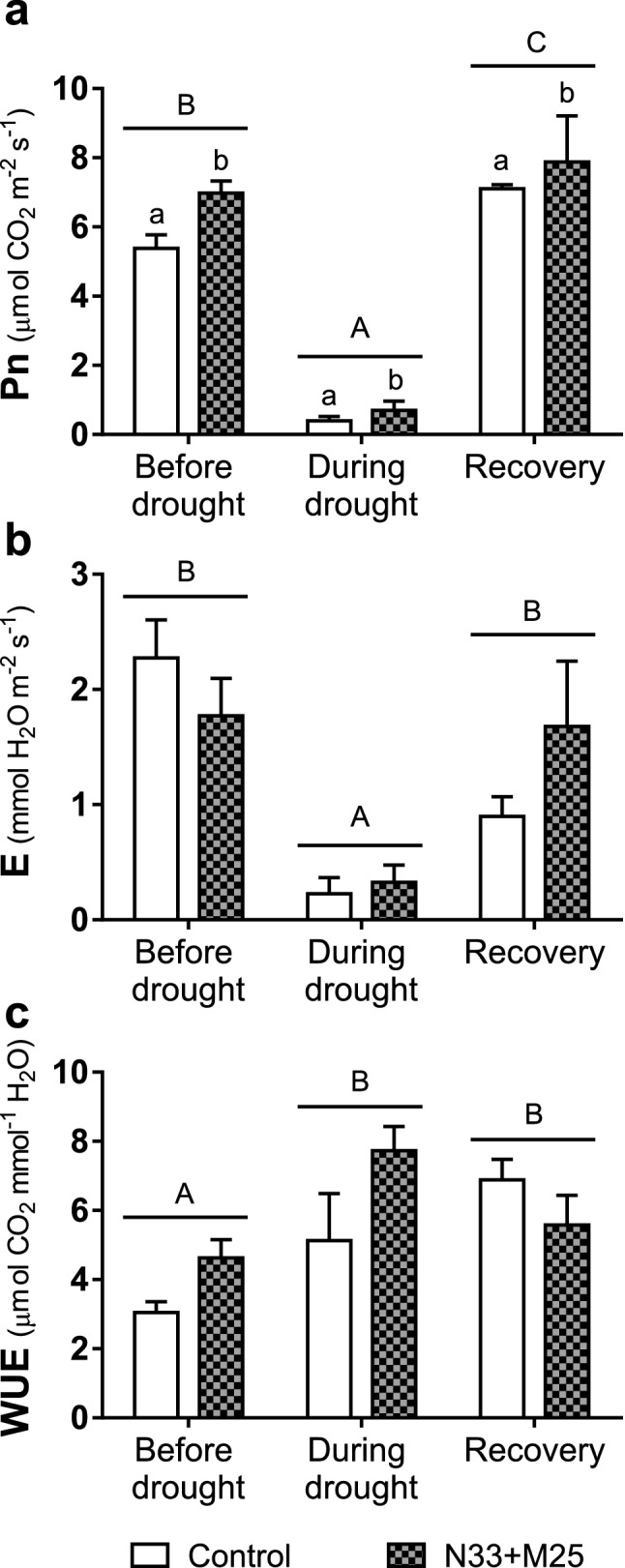
Evaluation of gas exchange parameters from *E. grandis* seedlings co-inoculated with N33 and M25 and subjected to rapid drought stress. Measurements were performed with an infrared gas analyzer at three different times: 50 DAT (before imposing the drought stress), 57 DAT (after 7 days without watering) and 74 DAT (recovery period, 14 days after the resumption of normal watering). The intrinsic water use efficiency (WUE) was calculated by dividing net photosynthesis (P_n_) and transpiration rate (E). Data shown are the means, n = 10, error bars indicate SEM. Different letters indicate significant differences in a two-way ANOVA (*P*-value < 0.05) for each parameter. Uppercase letters indicate the irrigation factor (before drought, during drought or recovery), while lowercase letters indicate the inoculation factor (non-inoculated or N33 + M25). The interaction between irrigation and inoculation factors was no significant in all cases.

We also monitored gas exchange parameters (P_n_, E, and intrinsic WUE) before, during and after drought stress (at 50, 57 and 74 DAT, respectively). The results showed that the co-inoculation with N33 and M25 stimulated P_n_ in *E. grandis* seedlings, reaching higher values than in non-inoculated plants, irrespective of the irrigation factor. On the other hand, no effect of bacteria was observed on E or intrinsic WUE. The suspension of irrigation caused a significant (*P*-value < 0.05) decrease of P_n_ and E in all plants, while the intrinsic WUE increased during the drought stress. With the resumption of irrigation, E fully recovered to similar rates as before drought, but plants showed a higher P_n_. As a consequence, both inoculated and non-inoculated plants maintained higher water use efficiencies even in the recovery period (Fig. 9).

### Retrieval of bacteria from non-sterile soil and from* E. grandis* seedlings after drought stress

To track the bacteria inside seedling tissue and to evaluate whether the inoculum was able to persist in planting soil in the absence of SAPH, bacterial strains were tagged with green fluorescent protein and inoculated in 0.5 l pots, containing two-month-old *E. grandis* seedlings and filled with non-sterile soil. Initially, the pots were watered frequently for 16 days to allow for microbe-plant-soil interaction under normal growth conditions. After that, watering was stopped for 2 weeks, resumed and maintained for 3 weeks. To assay strain survival, samples of inoculated and non-inoculated soil were collected regularly after initial inoculation. At the final time, rhizoplane, root and leaf samples were collected as well. Our results showed that after 50 days in a growth chamber, the inoculum can be retrieved from soil and rhizoplane, even after 14 days without watering (Supplementary Fig. S4). Strain M25 presented a higher survival titer than N33 (1 × 10^4^ and 1 × 10^3^ CFU per gram of fresh soil, respectively). We were unable to recover fluorescent bacteria from either inner root or leaf samples. We observed a similar bacterial rhizosphere growth and lack of colonization of plant inner tissues in drought experiments in which the seedlings had been grown in sand:vermiculite substrate (data not shown).

## Discussion

A good quality bioinoculant should be able to promote effective contact of the PGPB with the host plant, protecting the added PGPB from the natural environment and the competing native microbial community; also, its application should be simple and cost-effective^39^. Our work shows that the superabsorbent polymer developed by our research group (SAPH^12^), can be used as a vehicle to inoculate beneficial bacteria in the rhizosphere of eucalypt seedlings during transplantation. Moreover, the SAPH maintained the viability of the inoculum in soil for at least three months under laboratory conditions, which is the critical period for the establishment from greenhouses seedlings. Twelve weeks after formulation, we were able to re-isolate the two *Pseudomonas* strains from SAPH in large numbers (1 × 10^7^ CFU ml^−1^).

There is a positive influence of these bacteria in the management of water by *E. grandis* subjected to abiotic stress. This study shows that *Pseudomonas* strains N33 and M25 (individually or co-inoculated) can influence water management by stimulating plants to deploy an early response to water deficit and closing stomata. Decreasing g_s_ is a common strategy to avoid excessive water loss by transpiration in eucalypts^14,16,48^, including *E. grandis*^17,22^. This mechanism could be related to the higher RWC observed in leaves of inoculated plants after recovery from drought stress. M25-inoculated seedlings displayed higher RWC than non-inoculated plants, only after the recovery period in both gradual and rapid imposition of drought stress. Some PGPB can show their positive effect after rewatering even if they do not cause apparent effects during the water deficit period. For example, a considerably higher (5%) RWC was reported in tomato and pepper inoculated with *Achromobacter piechaudii*^32^, similar to the values obtained in the present work (2 and 7% for gradual and rapid drought, respectively). N33 showed intermediate values between control and M25 inoculated plants. Under normal watering, co-inoculated plants showed a 3% higher RWC than in non-inoculated plants, an observation also reported for other PGPB^35,49,50^. In our work, the fall on RWC due the water deficit was around 12 and 22% in rapid drought experiments, while in the gradual drought assay RWC decreased only 4%, even when pots had reached a minimal water content. This supports the idea that bacteria increase tolerance to gradual drought stress through an acclimation mechanism. Under sudden and severe water deficit, tree species may lose all their leaves^16^, while maintaining the capacity of resprouting and generating new leaves when water is provided again. In eucalypts, these types of plastic responses have been shown to depend on hydraulic properties^14,48^. When drought stress was imposed rapidly in our growth chamber assay, we observed a severe defoliation followed by a rapid recover of leaf area after rewatering. By contrast, under gradual and progressive drought stress, trees of the *Eucalyptus* genus can acclimate to water deficit without sacrificing leaves and reorganizing leaf area, through changes in hydric balance and water economy^14^. In our greenhouse drought assays, where stress was applied gradually, plants suffered only slight defoliation, but changed hydraulic parameters so as to adjust to the situation. M25-inoculated plants in the greenhouse accumulated more water in the apoplastic fraction than non-inoculated eucalypts, regardless of the irrigation condition. Moreover, they presented a lower modulus of the E_max_ coefficient, which is related to the elasticity of cell walls. More elastic cells allow seedlings to handle hydric stress, helping to maintain tissue turgor with lower RWC^19,51^.Therefore, this strain appears to have turned plants more conservative in their use of water, since M25-inoculated plants responded by developing leaves with more elastic cell walls and higher apoplastic water content and were faster than control plants in closing their stomata after the onset of drought stress.

Prolonged drought stress leads to stomatal closure and can damage chlorophyll and photosystems, causing a decreased photosynthetic capacity^52^. Modification of gas exchange to potentiate photosynthesis is another of the frequent effects of many PGPB^31^, which might contribute to the maintenance of productivity under water deficit by these microorganisms^34,35^. We observed positive effects of bacteria on the modulation of gas exchange parameters. When inoculated individually, P_n_ fell during the drought stress, without differences between inoculated or non-inoculated eucalypts, but it reached higher values in inoculated plants during the recovery phase. For the co-inoculation assay, P_n_ of inoculated plants was higher than in control plants during drought and recovery and even before imposing the drought.

Moreover, N33-inoculated plants showed a positive trend towards growth stimulation under well-watered conditions in the growth chamber assay. Both N33 and M25 caused an increase in height and collar diameter on *E. grandis* seedlings after a drought period, which only was statistically significant when both strains were co-inoculated. This could be due to a synergism between bacteria when applied together^35^. Eucalypt growth was noticeably faster in the greenhouse, probably due to the higher amount of light that plants received. In this case, gradual water stress caused slight defoliation but as inoculated plants built leaves with higher cell wall elasticity, they were better able to withstand stress, and when watering was resumed, inoculated plants grew more than non-inoculated ones, in spite of having similar leaf area. In the rapid water deficit, N33 and M25 stimulated plants to create new leaves when drought finished, resulting in a higher final leaf area and a slight increase in growth compared with the non-inoculated plants. However, when both bacteria were co-inoculated in a rapid-drought experimental design, inoculation did not increase the leaf area but significantly increased plant growth. In agreement with these results, synergic effects of different PGPB were observed in many herbaceous species, with fewer studies in arboreal species^30,34^.

In summary, we showed that the SAPH superabsorbent polymer can be used as a vehicle to apply beneficial bacteria to tree seedlings during transplantation and we identified two *Pseudomonas* strains that can confer better growth performance to the seedlings, under normal watering conditions and when subjected to water deficit. The combination of SAPH and beneficial bacteria constitutes an environment-friendly alternative to synthetic stimulants.

## Materials and methods

### Source of bacterial strains, screening, selection and tagging with GFP

The soil and plant-associated bacteria that we screened for enhancement of the germination rate of *Eucalyptus grandis* seeds came from several sources. *Pseudomonas protegens* CHAO (formerly designated as *Pseudomonas fluorescens* CHAO) is the type strain of this species^47^ and is available in public culture collections (CFBP 6595, DSM 19095). Strains BT2, BT4, BT5, GT4, MT3, MT8, NT2, NT3, NT6 and N1-N47 were originally isolated from leaves of tomato and other greenhouse-grown horticultural crops in La Plata district, Argentina^45^. *Bacillus amyloliquefaciens* ARP_2_3 and MEP_2_18, *Bacillus velezensis* A6 and *Bacillus subtilis* A7 were kindly provided by Dr Edgardo Jofré (*Departamento de Ciencias Naturales, Facultad de Ciencias Exactas, Físico Químicas y Naturales, Universidad Nacional de Río Cuarto, Argentina*). These strains were obtained from agricultural soils (*Río Cuarto*, *Córdoba* Province, Argentina) and were characterized in previous works^44,46^. *Pantoea eucalypti* M91 and *Pseudomonas* sp M22 and M25 were isolated from the rhizosphere of narrow-leaf birdsfoot trefoil (*Lotus tenuis*) plants native from the Salado River Basin (*Buenos Aires* Province, Argentina)^43^. The taxonomic identity of strain N33 was estimated on the basis of 16SrRNA gene sequence analysis. For this purpose, total genomic DNA was extracted and purified using the AccuPrep Genomic DNA Extraction Kit (*Bionner*, USA), according to the manufacturer’s instructions. Nearly full-length 16S rRNA gene was amplified using the universal primers 41f. (5-gCTCAAgATTgAACgCTggCg-3) and 1488r (5-ggTTACCTTgTTACgACTTCACC-3), as described previously^43^. The amplicons were sequenced (Macrogen, Korea) and aligned with reference sequences obtained from The Ribosomal Database Project Release 11.5^53^. The nucleotide sequence of 16S rRNA was deposited in the GenBank Database under accession number MT792080.

Bacterial inoculums were prepared by growing each strain overnight in 5 ml of liquid LB medium at 28 ºC on a rotator shaker (180 rpm). The bacterial suspensions were centrifuged (2000 rcf, 10 min) and resuspended in 10 mM MgCl_2_. Each suspension was adjusted to an OD_570_ of 0.1 (Sensident Scan plate reader, Merck, Darmstadt, Germany), corresponding to a concentration of 1 × 10^8^ colony-forming units (CFU) per milliliter. 50 mg of seed mixture (composed of seeds and floral remains and certified as containing approximately 600 viable seeds per gram of mixture; Paul Forestal, Entre Ríos, Argentina) were mixed with 1 ml of bacterial suspension for 30 min. Control seeds were mixed with 1 ml 10 mM MgCl_2_. The seeds were decanted and spread on Petri plates with absorbent paper wetted with 10 ml of mineral nutrient solution^54^. After seven days in darkness, the plates were exposed to light (250 μmol photon m^−2^ s^−1^, photoperiod 16/8 h L/D, 22 ± 2 °C, 65–70% RH) for three days and the number of emerged seedlings was counted on the tenth day since initial imbibitions, as we had previously determined that the germination rate in uninoculated seeds had stabilized by then.

*Pseudomonas sp.* strains M25 and N33 expressing GFP and kanamycin resistance were generated by four-parental mating conjugation using the mini-Tn7 transposon system^55^.

### Survival of bacterial strains embedded in SAPH

The acrylic-cellulosic polymer SAPH^12^ was hydrated with bacterial suspensions containing 1 × 10^8^ CFU ml^−1^ in distilled water. Control SAPH was hydrated with distilled water. Sixty ml of these inoculated and non-inoculated SAPHs were transferred to glass jars with aluminum lids that allowed gas exchange and kept in darkness in a growth chamber (22 ± 2 °C, 65–70% RH) for three months. Samples weighing 0.8–1.4 g of inoculated or non-inoculated SAPH were collected periodically and diluted serially. Fifty μl of each dilution were plated in duplicate onto LB-agar medium and the resulting fluorescent colonies were counted.

### Design of drought experiments

*E. grandis* seedlings were grown for two months in a growth chamber (250 μmol photon m^−2^ s^−1^, photoperiod 16/8 h L/D, 22 ± 2 °C, 65–70% RH) in 80 ml sand-filled pots and fertilized regularly. For the gradual drought experiment, which was carried out between February and June 2018, plants were moved to a greenhouse located in La Plata (Buenos Aires, − 34.912375, − 57.930637). The temperature ranged from 9.8 to 33.4 ºC, the photon flux density on sunny days was around 1000 μmol photon m^−2^ s^−1^ at midday, and the relative humidity was 50–70%. After a two-week acclimation, the seedlings were inoculated by transplanting to 10 l pots containing substrate (sand:vermiculite 1:1) together with 500 ml of previously hydrated SAPH containing 1 × 10^10^ CFU of bacterial cells. In the control group, SAPH was hydrated with distilled water. A bifactorial design was used, with “strain” and “drought” as main factors. Twelve plants per treatment were selected randomly. Plants were watered weekly with tap water to field capacity and supplemented with 250 ml of nutritive solution^54^. This regime was maintained for 35 days after transplant (DAT), after which all pots were covered with nylon bags closed at the plant collar and watering was suspended during 55 days for the “drought” group. During this period, the “well-watered” group continued to be watered weekly to field capacity. Then, all the plastic bags were removed and weekly watering was resumed for further 24 days (“recovery”). For the growth-chamber drought experiments, two-month-old seedlings were inoculated by transplanting to 0.5 l pots with sand:vermiculite (1:1) together with 150 ml of previously hydrated SAPH containing 1 × 10^10^ CFU of either strain bacterial. The same bifactorial design was used as in the greenhouse experiments, except that 8–10 plants per treatment were selected randomly. The watering was performed weekly to field capacity with tap water plus 150 ml of nutritive solution^54^, during 45 days after the transplant. Suspension of watering and recovery were similar to greenhouse experiment but lasted for 10 and 14 days, respectively. For the co-inoculation assay, the same procedure was followed, except that the inoculation was done with 150 ml SAPH containing 0.5 × 10^10^ CFU of each strain mixed together and the plants were left to grow for 50 days under normal watering before the drought started. In all experiments, plants were rotated regularly to avoid position effects.

### Growth parameters

Diameter at the collar and plant height were measured weekly, using a metric tape and a Vernier caliper. Plant height was measured from the substrate level to the main apex, with 0.5 cm intervals. The diameter at the collar was measured at the seedling base immediately above the first secondary root. All measurements are reported as increments relative to the initial value at inoculation time. The paired values from plants were used to calculate a “growth index” (GI) by the difference in the volumetric index, calculated at the beginning and end of the experiment by multiplying the height and the square of collar diameter (GI = diameter^2^.height). Stems, roots, and leaves of every plant were separated, dried and weighed to obtain the final organ biomass. The total leaf area per plant was calculated from a subgroup of leaves and the total dried leaf weight, at the final time. The leaf subgroups were scanned, their area was calculated using Photoshop version CS3 software (www.adobe.com), and then dried for 48 h at 60 ºC and weighed. The total leaf area was calculated as the total leaf biomass multiplied by the scanned area and divided by the leaf subgroup dry mass.

### Relative leaf water content (RWC)

A fully expanded leaf from the main stem of every plant was cut and weighed immediately to obtain the fresh weight (FW). Then, the leaves were submerged into distilled water for 24 h and re-weighed to compute the full-turgor weight (TW). After that, the leaves were dried at 60 ºC for 48 h to obtain their dry weight (DW). RWC was calculated as (FW-DW) / (TW-DW) × 100.

### Pressure–volume curves

For the greenhouse assay, one branch of each of 6 plants per treatment was cut at the end of the recovery period. The branches were hydrated with distilled water to maximum turgor and weighed. Water potential was measured with a Scholander pressure chamber (Biocontrol, Buenos Aires, Argentina). The branches were left to dehydrate and their weight and water potential were measured periodically until complete dehydration. The resulting data pairs were plotted and analyzed with the macro available at https://landflux.org/Tools.php, based on published methodology to adjust the pressure–volume curves to a biphasic linear model^56^. The informative points extracted from curve fitting were: Ψo = osmotic potential at inner water saturation; Ψo TLP = osmotic potential at the turgor loss point; apoplastic RWC = relative water content in the apoplast and E_max_ = coefficient of maximum elasticity.

### Gas exchange parameters

Stomatal conductance was measured between 10 am and 16 pm with a steady-state diffusion porometer (SC-1 Decagon Devices, Pullman, Washington) on the youngest fully expanded leaf of the main stem. The same criterion was used to select the leaves for gas exchange measurements at light saturation with an infrared gas analyzer (LI6400, LI-COR, Lincoln, NE). This allowed us to make measurements in all plants, even when individual leaves had fallen. Leaves were equilibrated in the gas-exchange chamber at 400 ppm CO_2_ and illuminated with photosynthetically active radiation of 1200 μmol m^−2^ s^−1^ provided by a red/blue LED light source. Temperature, relative humidity, and airflow rate inside the chamber were maintained at 22 °C, 65%, and 500 mmol s^−1^, respectively. Instantaneous water use efficiency was calculated as the ratio between net photosynthesis and transpiration rate.

### Recovery of bacteria from nonsterile soil and *E. grandis* seedlings after drought stress

Two-month old *E. grandis* seedlings were grown in 0.5 l pots, filled with non-sterile commercial soil (Grow Mix Multipro, Terrafertil, Buenos Aires). Cultures of M25 and N33 strains expressing GFP were resuspended in distilled water and potted seedlings were inoculated by watering with 100 ml of the bacterial suspension (1 × 10^8^ CFU ml^−1^); control seedlings were watered with distilled water. Plants were left to grow normally for 16 days to allow interaction with inoculated bacteria and other microorganisms of the non-sterile soil. Then, watering was suspended for 14 days, followed by 21 days of recovery (51 days total). Soil samples were collected periodically. After recovery, rhizoplane (i.e., soil near the root surface), root and leaf samples were collected, surface-sterilized, washed with distilled water and extracted by grinding with 10 mM MgCl_2_. The resulting suspension was filtered to remove debris, the supernatant was diluted serially and used to plate LB agar medium, supplemented with 50 μg ml^−1^ of kanamycin. The fluorescent colonies obtained were counted.

### Statistical analysis

Selection of bacteria strains was analyzed with 95% confidence intervals. Data from drought assays were evaluated by using the means of each parameter in a one-way or two-way ANOVA, as appropriate, with α = 0.05 in all cases. Homocedasticity and normality were checked before ANOVA. When heteroscedasticity was detected, the variance was modelled with General Linear Models (GLM). In two-factor ANOVA and GLM, if the interaction between factors was significant (*P*-value < 0.05), the Tukey post-hoc analysis was done comparing all possible treatment combinations (results indicated in the figures and tables with lowercase letters). In the case only main factors were significant but not the interaction, the means of the levels of those factors were compared (uppercase letters for irrigation factor and lowercase letters for inoculation factor). All analyses were performed with the software InfoStat^57^ and data were plotted with GraphPad Prism version 6.01 for Windows, GraphPad Software, La Jolla California USA, www.graphpad.com.

## Supplementary information

Supplementary Information

## Data Availability

The data and materials that support the findings of this study are available from the corresponding author upon request.
