# Supplementary material for: Growth promotion and protection from drought in Eucalyptus grandis seedlings inoculated with beneficial bacteria embedded in a superabsorbent polymer
Source: Sci Rep. 2020 Oct 26;10:18221. doi: 10.1038/s41598-020-75212-4 (PMC7588442; doi:10.1038/s41598-020-75212-4)

Growth promotion and protection from drought in *Eucalyptus grandis* seedlings inoculated with beneficial bacteria embedded in a superabsorbent polymer

José María Chaín, Esteban Tubert, Corina Graciano, Luis Nazareno Castagno, Marina Recchi, Fernando L. Pieckenstain, María Julia Estrella, Gustavo Gudesblat, Gabriela Amodeo, Irene Baroli

Supplementary figures

Supplementary Figure S1: Biomass allocation computed the final time of drought assays.

Supplementary Figure S2: Estimation of Leaf Area Index (LAI) as incident light interception in *E. grandis* seedlings subjected to rapid drought stress.

Supplementary Figure S3: Growth index comparison between greenhouse and growth chamber assays at the same time after transplant.

Supplementary Figure S4: Localization of the selected bacteria after drought stress *in E. grandis* seedlings grown in nonsterile soil.

Supplementary Figure S1

Biomass allocation computed the final time of drought assays. Well-watered (WW) plants were maintained watered to field capacity. Plants that were stressed by suspending the irrigation and recovered when watering was resumed are indicated as D + R. All bars represent means ± SEM (n=8-12). Different letters above the bars indicate significant differences in a two-way ANOVA (P-value < 0.05). Uppercase letters indicate the irrigation factor (Well-Watered or Drought + Recovery). Lowercase letters indicate the interaction between inoculation (non-inoculated, N33 or M25) and irrigation factors. Lack of letters indicates non-significant differences in the main factors (irrigation and inoculation) or factor interaction (P-value > 0.05).

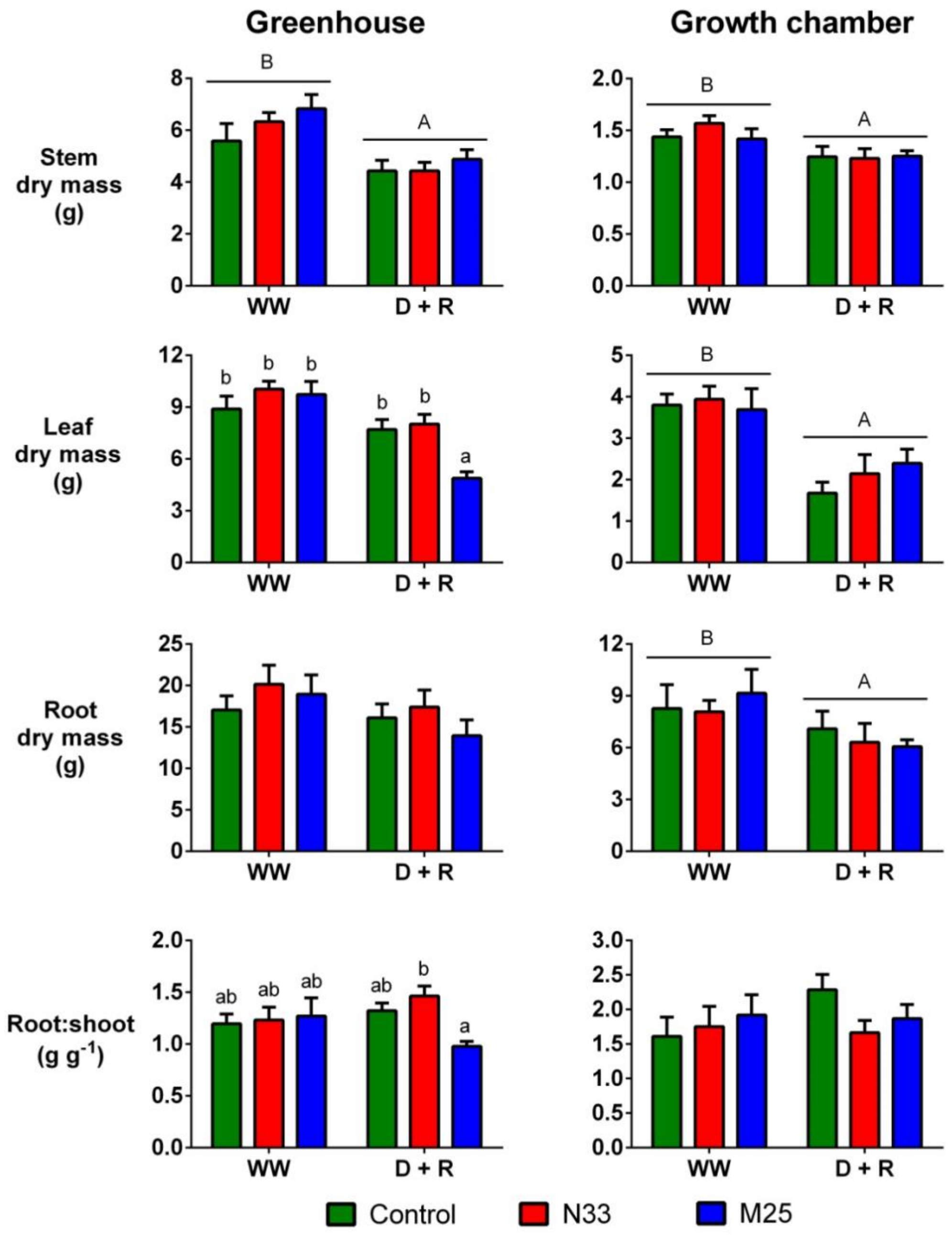

Supplementary Figure S2

Estimation of Leaf Area Index (LAI) as incident light interception in *E. grandis* seedlings subjected to rapid drought stress. Water regime for stressed plants (D+R) is indicated above of each panel. Well watered plants (WW) were watered every other day to field capacity. (a) Light interception across all experiment duration. (b) Zoom of the graph (a) only showing drought and recovery period. DAT = days after transplant. Data shown are the means, n = 4, error bars represent SEM.

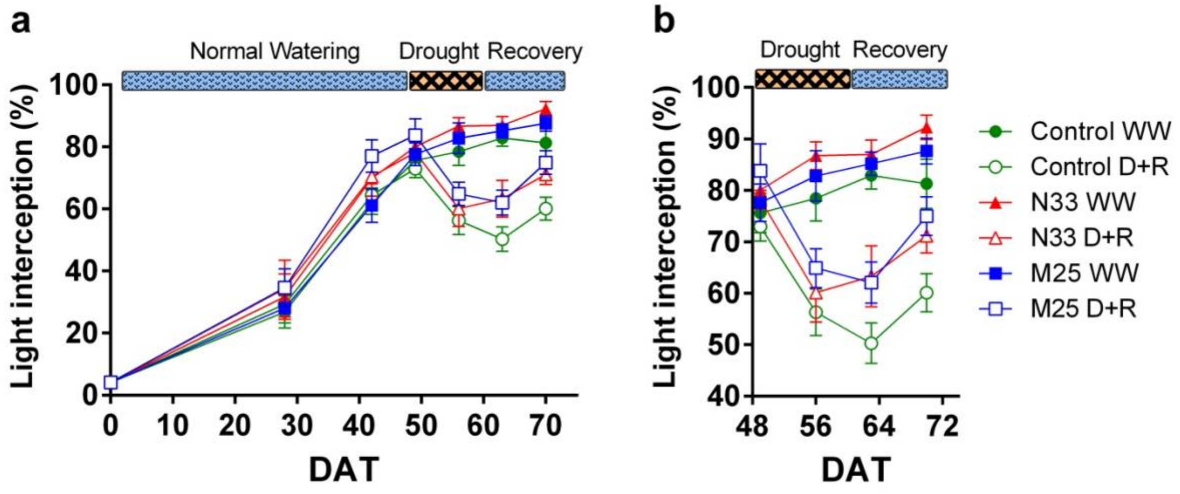

Supplementary Figure S3

Growth index comparison between greenhouse and growth chamber assays at the same time after transplant (final time for growth chamber experiment)**.** All bars represent means ± SEM (n=8-12). The smooth bars indicate plants that were maintained watered to field capacity (WW = Well watered plants). The striped bars indicate plants that were stressed by suspending the irrigation and recovered when watering was resumed (D+R = Drought plus recovery).

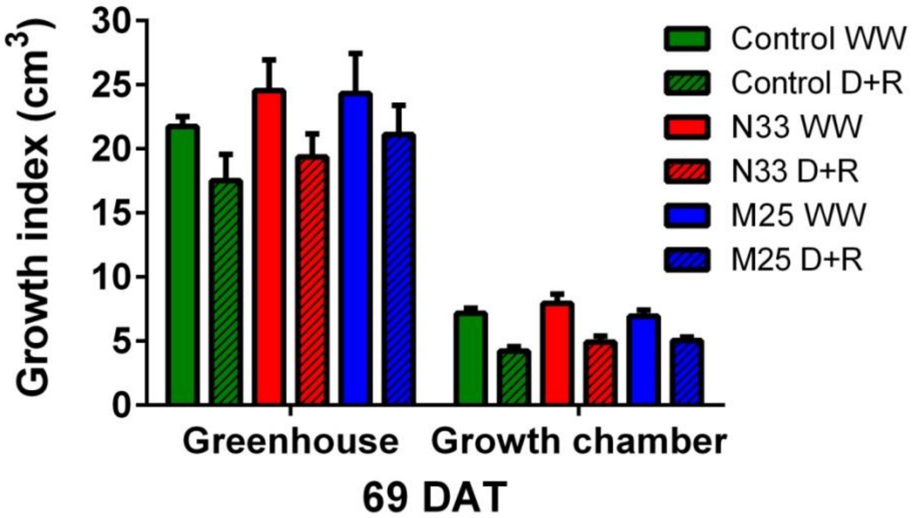

Supplementary Figure S4

Localization of the selected bacteria after drought stress *in E. grandis* seedlings grown in nonsterile soil. Soil, rhizoplane, root and leaf samples were processed to recover the inoculum. The resulting suspension was diluted serially and 50 µl of each dilution were plated on rich agar medium, as shown in the upper-left image.

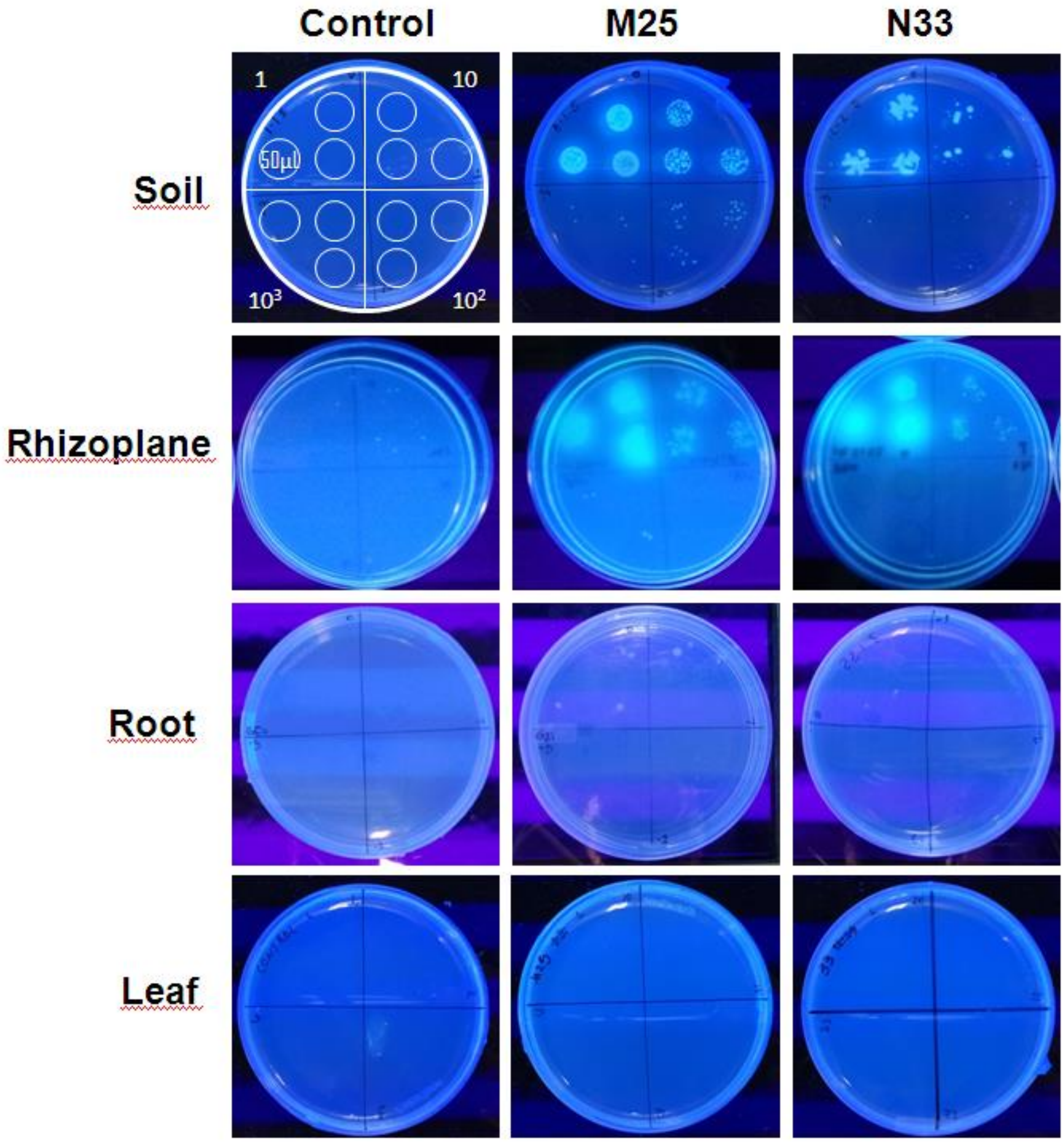

Supplement: Supplementary file 1 — Supplementary Information [file 41598_2020_75212_MOESM1_ESM.pdf]
